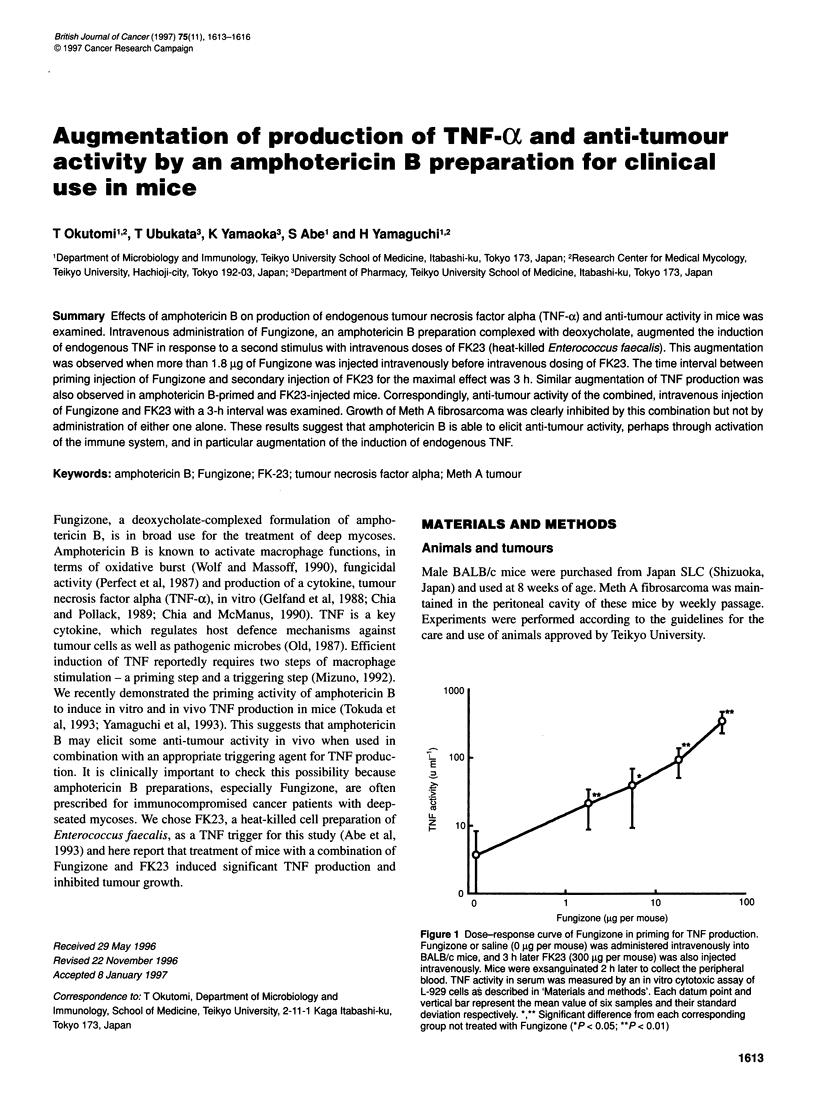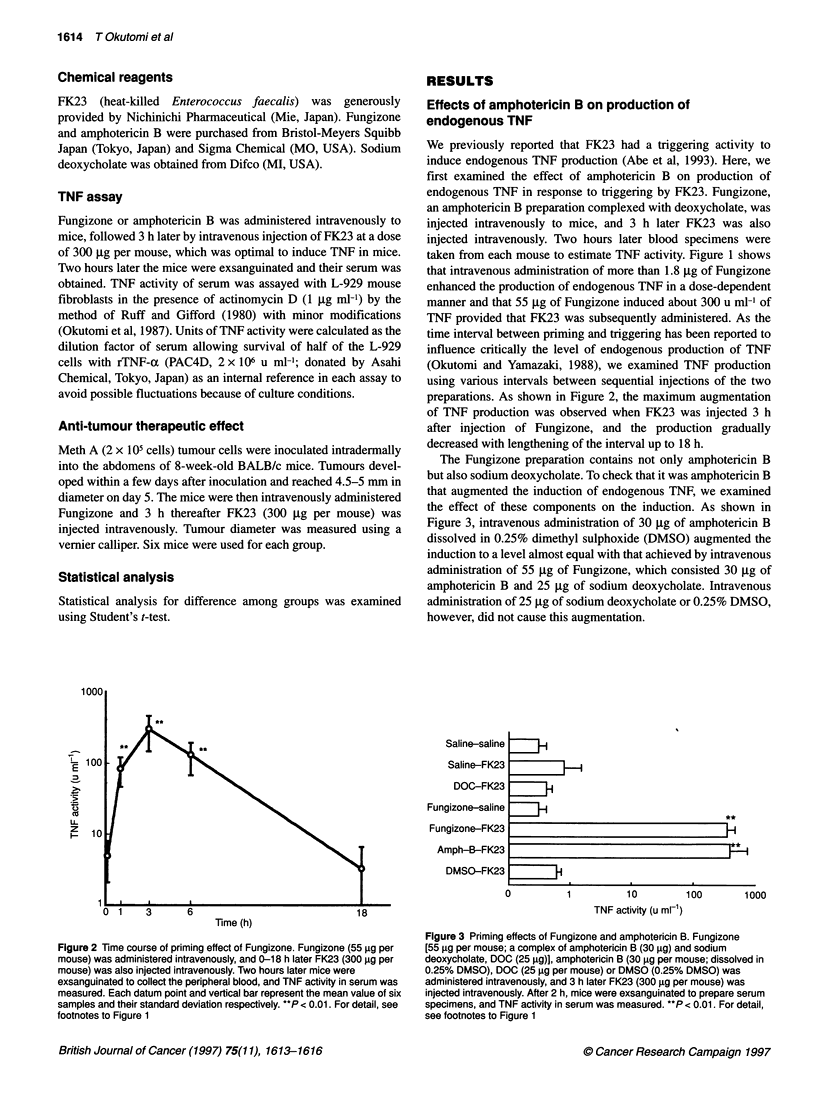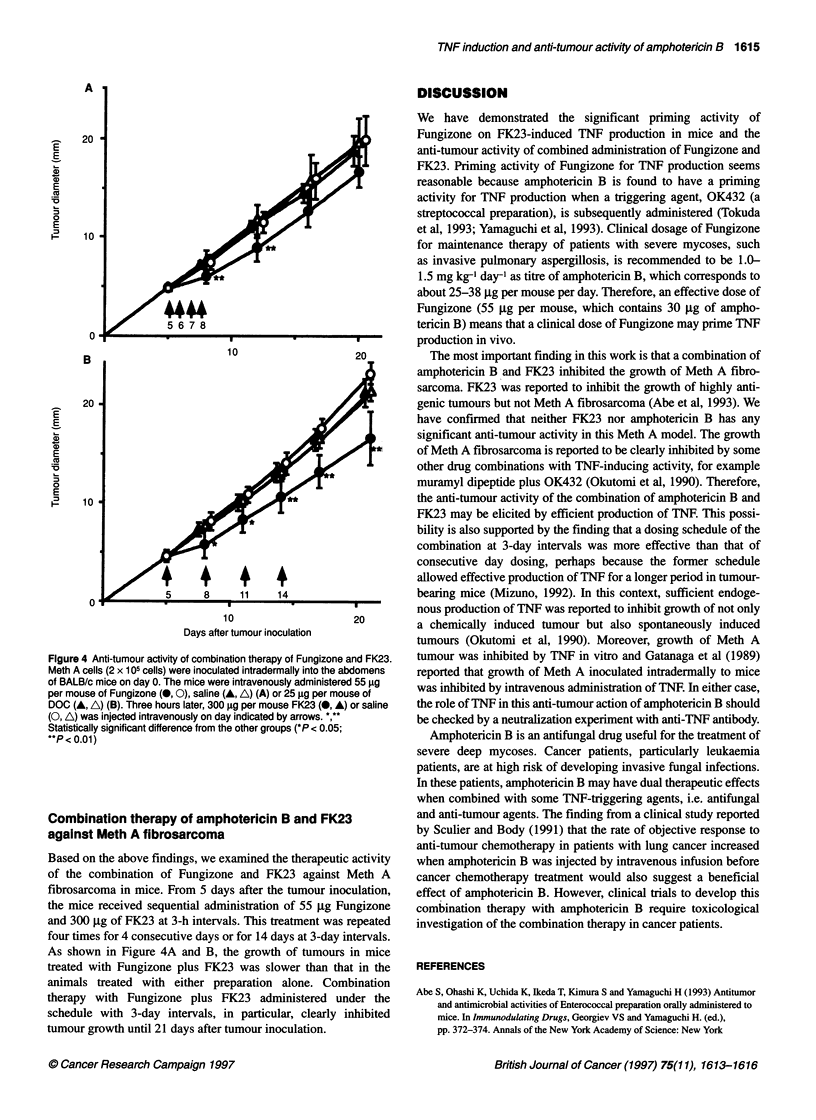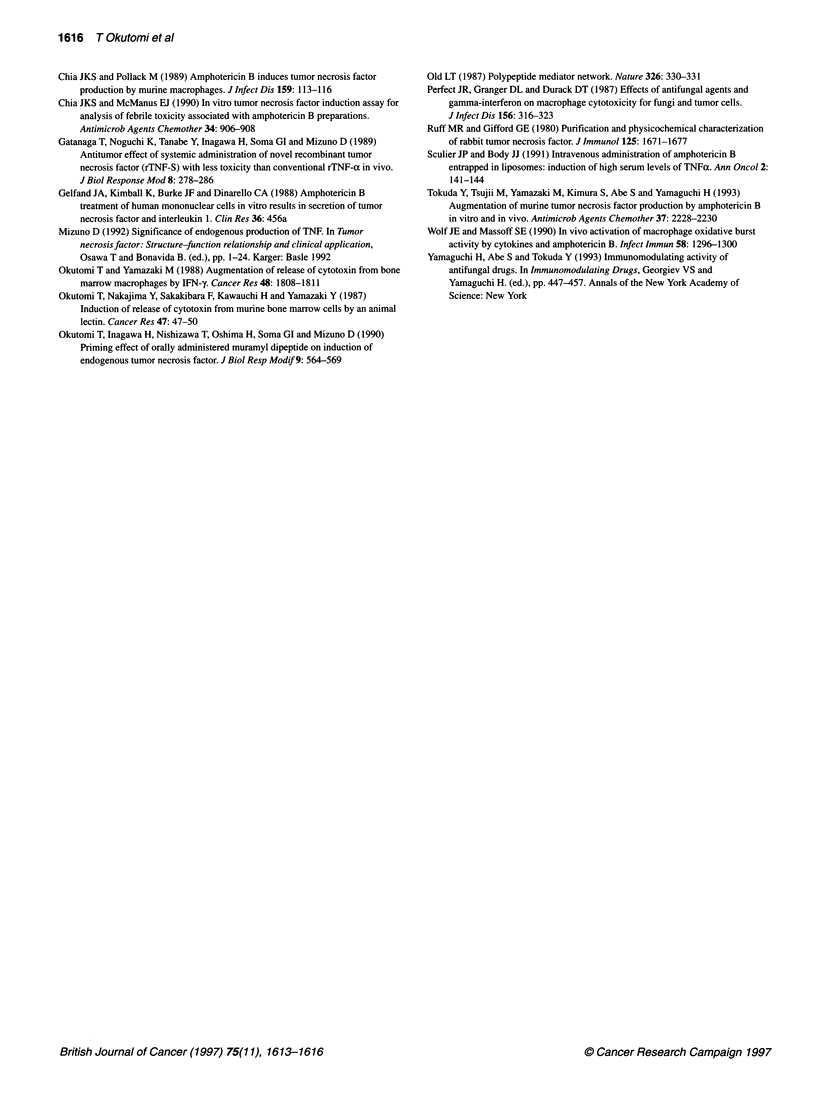# Augmentation of production of TNF-alpha and anti-tumour activity by an amphotericin B preparation for clinical use in mice.

**DOI:** 10.1038/bjc.1997.275

**Published:** 1997

**Authors:** T. Okutomi, T. Ubukata, K. Yamaoka, S. Abe, H. Yamaguchi

**Affiliations:** Department of Microbiology and Immunology, Teikyo University School of Medicine, Itabashi-ku, Tokyo, Japan.

## Abstract

Effects of amphotericin B on production of endogenous tumour necrosis factor alpha (TNF-alpha) and anti-tumour activity in mice was examined. Intravenous administration of Fungizone, an amphotericin B preparation complexed with deoxycholate, augmented the induction of endogenous TNF in response to a second stimulus with intravenous doses of FK23 (heat-killed Enterococcus faecalis). This augmentation was observed when more than 1.8 microg of Fungizone was injected intravenously before intravenous dosing of FK23. The time interval between priming injection of Fungizone and secondary injection of FK23 for the maximal effect was 3 h. Similar augmentation of TNF production was also observed in amphotericin B-primed and FK23-injected mice. Correspondingly, anti-tumour activity of the combined, intravenous injection of Fungizone and FK23 with a 3-h interval was examined. Growth of Meth A fibrosarcoma was clearly inhibited by this combination but not by administration of either one alone. These results suggest that amphotericin B is able to elicit anti-tumour activity, perhaps through activation of the immune system, and in particular augmentation of the induction of endogenous TNF.


British Journal of Cancer (1997) 75(11), 1613–1616
© 1997 Cancer Research Campaign

### Augmentation of production of TNF-α and anti-tumour activity by an amphotericin B preparation for clinical use in mice

**T Okutomi[1,2], T Ubukata[3], K Yamaoka[3], S Abe[1] and H Yamaguchi[1,2]**

[1]Department of Microbiology and Immunology, Teikyo University School of Medicine, Itabashi-ku, Tokyo 173, Japan; [2]Research Center for Medical Mycology, Teikyo University, Hachioji-city, Tokyo 192-03, Japan; [3]Department of Pharmacy, Teikyo University School of Medicine, Itabashi-ku, Tokyo 173, Japan

**Summary** Effects of amphotericin B on production of endogenous tumour necrosis factor alpha (TNF-α) and anti-tumour activity in mice was examined. Intravenous administration of Fungizone, an amphotericin B preparation complexed with deoxycholate, augmented the induction of endogenous TNF in response to a second stimulus with intravenous doses of FK23 (heat-killed *Enterococcus faecalis*). This augmentation was observed when more than 1.8 μg of Fungizone was injected intravenously before intravenous dosing of FK23. The time interval between priming injection of Fungizone and secondary injection of FK23 for the maximal effect was 3 h. Similar augmentation of TNF production was also observed in amphotericin B-primed and FK23-injected mice. Correspondingly, anti-tumour activity of the combined, intravenous injection of Fungizone and FK23 with a 3-h interval was examined. Growth of Meth A fibrosarcoma was clearly inhibited by this combination but not by administration of either one alone. These results suggest that amphotericin B is able to elicit anti-tumour activity, perhaps through activation of the immune system, and in particular augmentation of the induction of endogenous TNF.

**Keywords:** amphotericin B; Fungizone; FK-23; tumour necrosis factor alpha; Meth A tumour

Fungizone, a deoxycholate-complexed formulation of amphotericin B, is in broad use for the treatment of deep mycoses. Amphotericin B is known to activate macrophage functions, in terms of oxidative burst (Wolf and Massoff, 1990), fungicidal activity (Perfect et al, 1987) and production of a cytokine, tumour necrosis factor alpha (TNF-α), in vitro (Gelfand et al, 1988; Chia and Pollack, 1989; Chia and McManus, 1990). TNF is a key cytokine, which regulates host defence mechanisms against tumour cells as well as pathogenic microbes (Old, 1987). Efficient induction of TNF reportedly requires two steps of macrophage stimulation – a priming step and a triggering step (Mizuno, 1992). We recently demonstrated the priming activity of amphotericin B to induce in vitro and in vivo TNF production in mice (Tokuda et al, 1993; Yamaguchi et al, 1993). This suggests that amphotericin B may elicit some anti-tumour activity in vivo when used in combination with an appropriate triggering agent for TNF production. It is clinically important to check this possibility because amphotericin B preparations, especially Fungizone, are often prescribed for immunocompromised cancer patients with deep-seated mycoses. We chose FK23, a heat-killed cell preparation of *Enterococcus faecalis*, as a TNF trigger for this study (Abe et al, 1993) and here report that treatment of mice with a combination of Fungizone and FK23 induced significant TNF production and inhibited tumour growth.

*Received 29 May 1996*
*Revised 22 November 1996*
*Accepted 8 January 1997*

*Correspondence to:* T Okutomi, Department of Microbiology and Immunology, School of Medicine, Teikyo University, 2-11-1 Kaga Itabashi-ku, Tokyo 173, Japan

### MATERIALS AND METHODS

### Animals and tumours

Male BALB/c mice were purchased from Japan SLC (Shizuoka, Japan) and used at 8 weeks of age. Meth A fibrosarcoma was maintained in the peritoneal cavity of these mice by weekly passage. Experiments were performed according to the guidelines for the care and use of animals approved by Teikyo University.

**Figure 1** Dose–response curve of Fungizone in priming for TNF production. Fungizone or saline (0 μg per mouse) was administered intravenously into BALB/c mice, and 3 h later FK23 (300 μg per mouse) was also injected intravenously. Mice were exsanguinated 2 h later to collect the peripheral blood. TNF activity in serum was measured by an in vitro cytotoxic assay of L-929 cells as described in 'Materials and methods'. Each datum point and vertical bar represent the mean value of six samples and their standard deviation respectively. *,** Significant difference from each corresponding group not treated with Fungizone (*$P < 0.05$; **$P < 0.01$)

### Chemical reagents

FK23 (heat-killed *Enterococcus faecalis*) was generously provided by Nichinichi Pharmaceutical (Mie, Japan). Fungizone and amphotericin B were purchased from Bristol-Meyers Squibb Japan (Tokyo, Japan) and Sigma Chemical (MO, USA). Sodium deoxycholate was obtained from Difco (MI, USA).

### TNF assay

Fungizone or amphotericin B was administered intravenously to mice, followed 3 h later by intravenous injection of FK23 at a dose of 300 µg per mouse, which was optimal to induce TNF in mice. Two hours later the mice were exsanguinated and their serum was obtained. TNF activity of serum was assayed with L-929 mouse fibroblasts in the presence of actinomycin D (1 µg $ml^{-1}$) by the method of Ruff and Gifford (1980) with minor modifications (Okutomi et al, 1987). Units of TNF activity were calculated as the dilution factor of serum allowing survival of half of the L-929 cells with rTNF-α (PAC4D, $2 \times 10^6$ u $ml^{-1}$; donated by Asahi Chemical, Tokyo, Japan) as an internal reference in each assay to avoid possible fluctuations because of culture conditions.

### Anti-tumour therapeutic effect

Meth A ($2 \times 10^5$ cells) tumour cells were inoculated intradermally into the abdomens of 8-week-old BALB/c mice. Tumours developed within a few days after inoculation and reached 4.5–5 mm in diameter on day 5. The mice were then intravenously administered Fungizone and 3 h thereafter FK23 (300 µg per mouse) was injected intravenously. Tumour diameter was measured using a vernier calliper. Six mice were used for each group.

### Statistical analysis

Statistical analysis for difference among groups was examined using Student's *t*-test.

### RESULTS

### Effects of amphotericin B on production of endogenous TNF

We previously reported that FK23 had a triggering activity to induce endogenous TNF production (Abe et al, 1993). Here, we first examined the effect of amphotericin B on production of endogenous TNF in response to triggering by FK23. Fungizone, an amphotericin B preparation complexed with deoxycholate, was injected intravenously to mice, and 3 h later FK23 was also injected intravenously. Two hours later blood specimens were taken from each mouse to estimate TNF activity. Figure 1 shows that intravenous administration of more than 1.8 µg of Fungizone enhanced the production of endogenous TNF in a dose-dependent manner and that 55 µg of Fungizone induced about 300 u $ml^{-1}$ of TNF provided that FK23 was subsequently administered. As the time interval between priming and triggering has been reported to influence critically the level of endogenous production of TNF (Okutomi and Yamazaki, 1988), we examined TNF production using various intervals between sequential injections of the two preparations. As shown in Figure 2, the maximum augmentation of TNF production was observed when FK23 was injected 3 h after injection of Fungizone, and the production gradually decreased with lengthening of the interval up to 18 h.

The Fungizone preparation contains not only amphotericin B but also sodium deoxycholate. To check that it was amphotericin B that augmented the induction of endogenous TNF, we examined the effect of these components on the induction. As shown in Figure 3, intravenous administration of 30 µg of amphotericin B dissolved in 0.25% dimethyl sulphoxide (DMSO) augmented the induction to a level almost equal with that achieved by intravenous administration of 55 µg of Fungizone, which consisted 30 µg of amphotericin B and 25 µg of sodium deoxycholate. Intravenous administration of 25 µg of sodium deoxycholate or 0.25% DMSO, however, did not cause this augmentation.

**Figure 2** Time course of priming effect of Fungizone. Fungizone (55 µg per mouse) was administered intravenously, and 0–18 h later FK23 (300 µg per mouse) was also injected intravenously. Two hours later mice were exsanguinated to collect the peripheral blood, and TNF activity in serum was measured. Each datum point and vertical bar represent the mean value of six samples and their standard deviation respectively. **$P < 0.01$. For detail, see footnotes to Figure 1

**Figure 3** Priming effects of Fungizone and amphotericin B. Fungizone [55 µg per mouse; a complex of amphotericin B (30 µg) and sodium deoxycholate, DOC (25 µg)], amphotericin B (30 µg per mouse; dissolved in 0.25% DMSO), DOC (25 µg per mouse) or DMSO (0.25% DMSO) was administered intravenously, and 3 h later FK23 (300 µg per mouse) was injected intravenously. After 2 h, mice were exsanguinated to prepare serum specimens, and TNF activity in serum was measured. **$P < 0.01$. For detail, see footnotes to Figure 1

© Cancer Research Campaign 1997

**Figure 4** Anti-tumour activity of combination therapy of Fungizone and FK23. Meth A cells (2 x $10^5$ cells) were inoculated intradermally into the abdomens of BALB/c mice on day 0. The mice were intravenously administered 55 µg per mouse of Fungizone (●, ○), saline (▲, △) (**A**) or 25 µg per mouse of DOC (▲, △) (**B**). Three hours later, 300 µg per mouse FK23 (●, ▲) or saline (○, △) was injected intravenously on day indicated by arrows. *,** Statistically significant difference from the other groups (*$P$ < 0.05; **$P$ < 0.01)

### Combination therapy of amphotericin B and FK23 against Meth A fibrosarcoma

Based on the above findings, we examined the therapeutic activity of the combination of Fungizone and FK23 against Meth A fibrosarcoma in mice. From 5 days after the tumour inoculation, the mice received sequential administration of 55 µg Fungizone and 300 µg of FK23 at 3-h intervals. This treatment was repeated four times for 4 consecutive days or for 14 days at 3-day intervals. As shown in Figure 4A and B, the growth of tumours in mice treated with Fungizone plus FK23 was slower than that in the animals treated with either preparation alone. Combination therapy with Fungizone plus FK23 administered under the schedule with 3-day intervals, in particular, clearly inhibited tumour growth until 21 days after tumour inoculation.

### DISCUSSION

We have demonstrated the significant priming activity of Fungizone on FK23-induced TNF production in mice and the anti-tumour activity of combined administration of Fungizone and FK23. Priming activity of Fungizone for TNF production seems reasonable because amphotericin B is found to have a priming activity for TNF production when a triggering agent, OK432 (a streptococcal preparation), is subsequently administered (Tokuda et al, 1993; Yamaguchi et al, 1993). Clinical dosage of Fungizone for maintenance therapy of patients with severe mycoses, such as invasive pulmonary aspergillosis, is recommended to be 1.0–1.5 mg $kg^{-1}$ $day^{-1}$ as titre of amphotericin B, which corresponds to about 25–38 µg per mouse per day. Therefore, an effective dose of Fungizone (55 µg per mouse, which contains 30 µg of amphotericin B) means that a clinical dose of Fungizone may prime TNF production in vivo.

The most important finding in this work is that a combination of amphotericin B and FK23 inhibited the growth of Meth A fibrosarcoma. FK23 was reported to inhibit the growth of highly antigenic tumours but not Meth A fibrosarcoma (Abe et al, 1993). We have confirmed that neither FK23 nor amphotericin B has any significant anti-tumour activity in this Meth A model. The growth of Meth A fibrosarcoma is reported to be clearly inhibited by some other drug combinations with TNF-inducing activity, for example muramyl dipeptide plus OK432 (Okutomi et al, 1990). Therefore, the anti-tumour activity of the combination of amphotericin B and FK23 may be elicited by efficient production of TNF. This possibility is also supported by the finding that a dosing schedule of the combination at 3-day intervals was more effective than that of consecutive day dosing, perhaps because the former schedule allowed effective production of TNF for a longer period in tumour-bearing mice (Mizuno, 1992). In this context, sufficient endogenous production of TNF was reported to inhibit growth of not only a chemically induced tumour but also spontaneously induced tumours (Okutomi et al, 1990). Moreover, growth of Meth A tumour was inhibited by TNF in vitro and Gatanaga et al (1989) reported that growth of Meth A inoculated intradermally to mice was inhibited by intravenous administration of TNF. In either case, the role of TNF in this anti-tumour action of amphotericin B should be checked by a neutralization experiment with anti-TNF antibody.

Amphotericin B is an antifungal drug useful for the treatment of severe deep mycoses. Cancer patients, particularly leukaemia patients, are at high risk of developing invasive fungal infections. In these patients, amphotericin B may have dual therapeutic effects when combined with some TNF-triggering agents, i.e. antifungal and anti-tumour agents. The finding from a clinical study reported by Sculier and Body (1991) that the rate of objective response to anti-tumour chemotherapy in patients with lung cancer increased when amphotericin B was injected by intravenous infusion before cancer chemotherapy treatment would also suggest a beneficial effect of amphotericin B. However, clinical trials to develop this combination therapy with amphotericin B require toxicological investigation of the combination therapy in cancer patients.

### REFERENCES

Abe S, Ohashi K, Uchida K, Ikeda T, Kimura S and Yamaguchi H (1993) Antitumor and antimicrobial activities of Enterococcal preparation orally administered to mice. In *Immunodulating Drugs*, Georgiev VS and Yamaguchi H. (ed.), pp. 372–374. Annals of the New York Academy of Science: New York

© Cancer Research Campaign 1997

Chia JKS and Pollack M (1989) Amphotericin B induces tumor necrosis factor production by murine macrophages. *J Infect Dis* **159**: 113–116

Chia JKS and McManus EJ (1990) In vitro tumor necrosis factor induction assay for analysis of febrile toxicity associated with amphotericin B preparations. *Antimicrob Agents Chemother* **34**: 906–908

Gatanaga T, Noguchi K, Tanabe Y, Inagawa H, Soma GI and Mizuno D (1989) Antitumor effect of systemic administration of novel recombinant tumor necrosis factor (rTNF-S) with less toxicity than conventional rTNF-α in vivo. *J Biol Response Mod* **8**: 278–286

Gelfand JA, Kimball K, Burke JF and Dinarello CA (1988) Amphotericin B treatment of human mononuclear cells in vitro results in secretion of tumor necrosis factor and interleukin 1. *Clin Res* **36**: 456a

Mizuno D (1992) Significance of endogenous production of TNF. In *Tumor necrosis factor: Structure–function relationship and clinical application*, Osawa T and Bonavida B. (ed.), pp. 1–24. Karger: Basle 1992

Okutomi T and Yamazaki M (1988) Augmentation of release of cytotoxin from bone marrow macrophages by IFN-γ. *Cancer Res* **48**: 1808–1811

Okutomi T, Nakajima Y, Sakakibara F, Kawauchi H and Yamazaki Y (1987) Induction of release of cytotoxin from murine bone marrow cells by an animal lectin. *Cancer Res* **47**: 47–50

Okutomi T, Inagawa H, Nishizawa T, Oshima H, Soma GI and Mizuno D (1990) Priming effect of orally administered muramyl dipeptide on induction of endogenous tumor necrosis factor. *J Biol Resp Modif* **9**: 564–569

Old LT (1987) Polypeptide mediator network. *Nature* **326**: 330–331

Perfect JR, Granger DL and Durack DT (1987) Effects of antifungal agents and gamma-interferon on macrophage cytotoxicity for fungi and tumor cells. *J Infect Dis* **156**: 316–323

Ruff MR and Gifford GE (1980) Purification and physicochemical characterization of rabbit tumor necrosis factor. *J Immunol* **125**: 1671–1677

Sculier JP and Body JJ (1991) Intravenous administration of amphotericin B entrapped in liposomes: induction of high serum levels of TNFα. *Ann Oncol* **2**: 141–144

Tokuda Y, Tsujii M, Yamazaki M, Kimura S, Abe S and Yamaguchi H (1993) Augmentation of murine tumor necrosis factor production by amphotericin B in vitro and in vivo. *Antimicrob Agents Chemother* **37**: 2228–2230

Wolf JE and Massoff SE (1990) In vivo activation of macrophage oxidative burst activity by cytokines and amphotericin B. *Infect Immun* **58**: 1296–1300

Yamaguchi H, Abe S and Tokuda Y (1993) Immunomodulating activity of antifungal drugs. In *Immunomodulating Drugs*, Georgiev VS and Yamaguchi H. (ed.), pp. 447–457. Annals of the New York Academy of Science: New York

*© Cancer Research Campaign 1997*